# Absorbing the Absorb experience—don’t let the concept fade away

**DOI:** 10.1007/s12471-019-01334-8

**Published:** 2019-10-18

**Authors:** R. Delewi, J. J. Piek

**Affiliations:** Department of Cardiology, Academic Medical Center, University of Amsterdam, Amsterdam, The Netherlands

Coronary artery disease is a major cause of morbidity and mortality in patients with diabetes mellitus. In our daily catheterisation laboratory practice, patients with diabetes mellitus represent more than 25% of those who undergo revascularisation. Patients with diabetes are not only more likely to have coronary artery disease, but also to have a more complex and diffuse illness. Atherosclerosis also tends to be more rapidly progressive. Moreover, the long-term results after revascularisation with percutaneous coronary intervention or coronary artery bypass graft surgery are worse in diabetic patients [1, 2].

Coronary revascularisation with contemporary drug-eluting stents (DES) is associated with better clinical outcomes compared with early-generation DES and bare-metal stents. However, there are ongoing risks of in-stent restenosis and thrombosis. It is hypothesised that this is due to persistent inflammation, impaired vasomotion and ongoing tissue growth within the stent frame. Other concerns regarding DES in the long term include permanent side branch occlusion and the lack of ability to place a bypass graft at the stented location.

Fully biodegradable stents, also referred to as bioresorbable scaffolds, have been developed in an effort to overcome these disadvantages of traditional stenting. The concept is that with these devices, the stent is in place long enough to protect against subacute thrombosis, recoil and early restenosis, but not long enough to experience the long-term shortcomings. Therefore, the use of these scaffolds is hoped to improve outcomes in high-risk patients who may need repeat percutaneous coronary intervention or coronary artery bypass graft surgery, such as patients with diabetes mellitus.

Randomised trials have compared the efficacy and safety of the Absorb bioresorbable scaffolds with everolimus-eluting metallic stents [3–9]. Unfortunately, as also summarised and confirmed in meta-analyses, the risk of target lesion failure and stent thrombosis was higher with the Absorb bioresorbable vascular scaffold (BVS) when compared with DES [10]. Underdeployement of the stent, incomplete lesion coverage and malapposition have been attributed to these unfavourable outcomes of this thick strut scaffold.

Hommels et al. present the results of their experience, across the Benelux, with the implantation of the Absorb bioresorbable vascular scaffolds in 150 diabetes mellitus patients with non-complex lesions [11]. The authors describe that in these non-complex lesions, there was a 100% successful device implantation. In previous randomised controlled trials, scaffold implantation was associated with longer procedure times, more contrast usage and more bailout percutaneous coronary interventions with standard DES. More importantly, the authors describe an acceptable safety as well as acceptable outcomes in the short term in this high-risk population.

However, the study is limited by the absence of a control group due to the non-randomised controlled design. Also, the experience is limited by a small sample size due to the stop in clinical utilisation of the Absorb BVS during the study due the disappointing results of the previously mentioned randomised controlled trials. Advanced implantation techniques, as well as modern and thinner struts in the resorbable scaffolds are probably needed.

However, the concept of bioabsorbable stenting still remains an attractive one. Particularly in young high-risk diabetes mellitus patients who are anticipated to undergo multiple revascularisation strategies in their lifetime. Which is why we should not write off the new scaffolds too soon. The development of modern DES also had higher rates of stent thrombosis in the initial stages. We therefore encourage not only the further development of bioabsorbable scaffolds with thin struts but also the search for the right population and right lesion type, so that the next generation of patients will benefit in the long term.
